# Data analysis of molecular dynamics simulation trajectories of β-sitosterol, sonidegib and cholesterol in smoothened protein with the CHARMM36 force field

**DOI:** 10.1016/j.dib.2020.106350

**Published:** 2020-09-29

**Authors:** Mohammad Tasyriq Che Omar

**Affiliations:** Biology Section, School of Distance Education, Universiti Sains Malaysia, Pulau Pinang 11800, Malaysia

**Keywords:** Smoothened protein, β-sitosterol, Smoothened antagonist, MD simulation, GROMACS, MMPBSA

## Abstract

Inactivation of smoothened protein (SMO) by the antagonists in SHH-driven cancer types is essential for inhibition of cancer progression. This article presents molecular dynamics (MD) trajectories of water solution of three protein-ligand complexes smoothened-β-sitosterol (SMO-BST), smoothened-sonidegib (SMO-SNG) and smoothened-cholesterol (SMO-CLR) using CHARMM36 and SPC/E water model combination. Additionally, the work presents the topologies and trajectories of GROMACS files that were employed to analyse the protein-ligand interaction types (PyContact) and binding energy calculation (g_mmpbsa). The data demonstrated that equilibrated models of SMO-SNG and SMO-CLR complexes showed crucial residues that almost similar for interaction and contribution energy as previously reported in laboratory setup (*in vitro*). Initial simulations confirmed the role of ARG451 and TRP535 in the dynamic regulation of SMO. These data then were used as a reference for understanding the molecular dynamics of SMO-BST complex and thus predicted its mechanism of action.

**Specifications Table**

**Table:** 

Subject	Biological Sciences
Specific subject area	Computational Molecular Biophysics
Type of data	Molecular Dynamics (MD) Simulations Table Figure Video (mp4)
How data were acquired	Classical all-atom (AA) MD simulation in the explicit solvent with GROMACS, PyContact, g_mmpbsa and VMD
Data format	Raw - molecular dynamics structures and trajectories – compressed GROMACS structure and trajectory files (.tpr and .xtc) Analysed - Interaction types and Binding energy
Parameters for data collection	CHARMM36 force field NVT ensemble at 300 K
Description of data collection	Data were obtained from molecular dynamics simulation ran on Ubuntu 18.4 LTE desktop with GROMACS software version 2018.1
Data source location	Institution: Biology Section, School of Distance Education, Universiti Sains Malaysia City/Town/Region: Pulau Pinang Country: Country: Malaysia
Data accessibility	With the article and via - PubChem ID number CID24775005 (SNG), CID222284 (BST) and CID5997 (CLR) Repository name: Mendeley DataData identification number: http://dx.doi.org/10.17632/v94vzbwzf3.1 Direct URL to data: https://data.mendeley.com/datasets/v94vzbwzf3/1.

## Value of the Data

• Data represent the usefulness of dynamic simulation in understanding the binding between β-sitosterol and smoothened protein.
• Data can benefit researchers or scientist in the field of structural biology and drug discovery.
• The flow of MD analysis reported here is suitable for screening the potential molecules before verifying by *in vitro* analysis.
• Analysed trajectories provide information regarding the important interacted residues that exhibits receptor agonistic or antagonistic profile.

## 1 Data Description

All raw data provided in this article were generated for molecular dynamics (MD) simulations of human Smoothened protein complexes either with β-sitosterol, sonidegib or cholesterol. Minimized docked model of complex SMO-CLR, SMO-SNG and SMO-BST as an initial coordinate file for simulation are provided in PDB files format. The CHARMM36 force field is used and complexes solvated with SPC/E water molecules for the production of MD simulations. Raw data then will be analysed using software tools such as PyContact and g_mmpbsa to discover the interaction types and binding energy. The raw data were deposited at the public repository Mendeley Data (https://data.mendeley.com/datasets/v94vzbwzf3/1). Supplementary videos (Figure S1-S3) of crucial residue pair interaction are supplemented with this article.

**Fig. 1:**
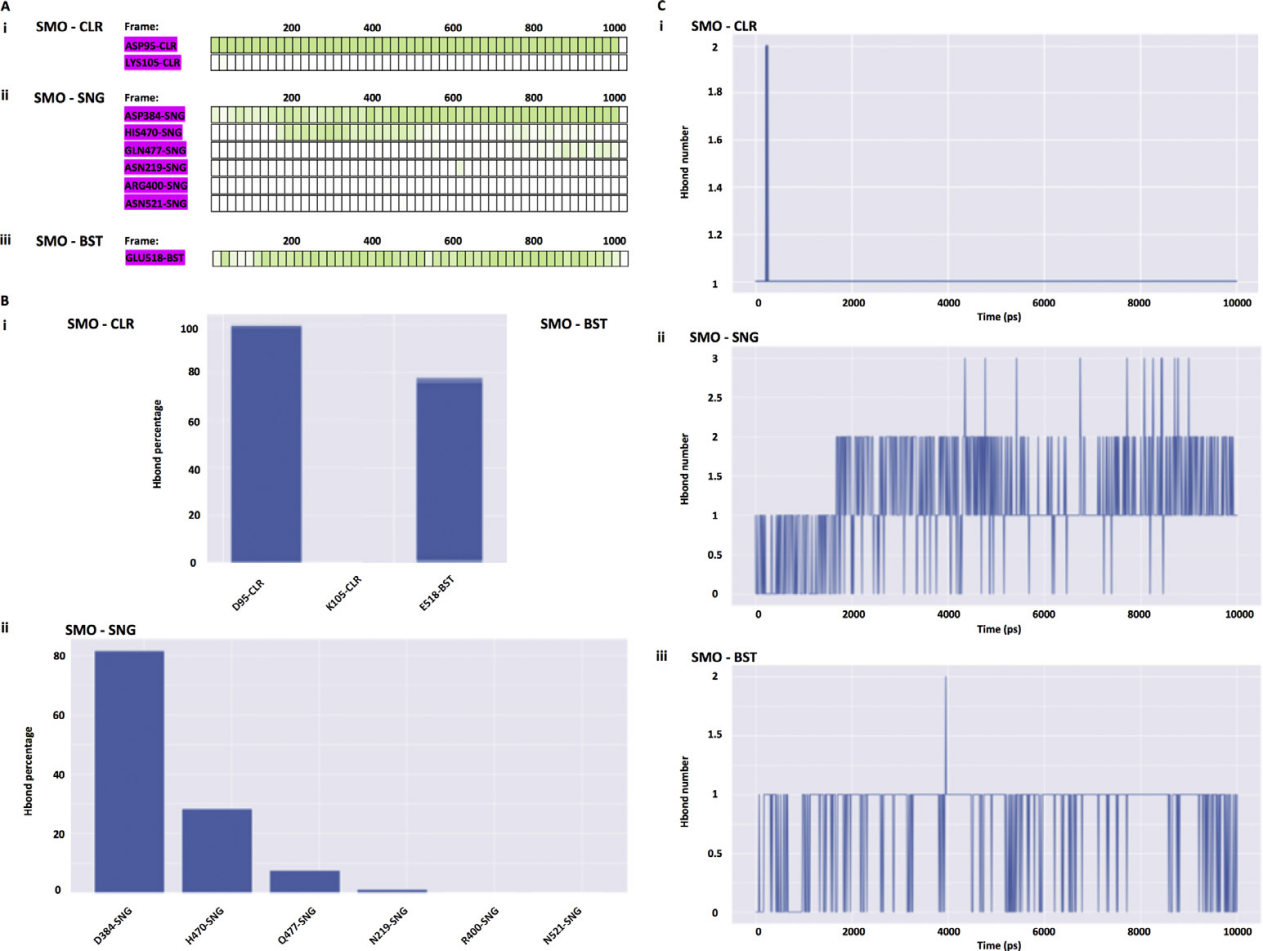
Hydrogen bond of smoothened protein (SMO) with cholesterol (CLR), sonidegib (SNG) and β-sitosterol (BST). A) Timeline of hydrogen bond formation (pink highlighted) of complexes throughout trajectories frames. Green boxes represent side-chain side-chain interaction B) Residues are responsible for the formation of a hydrogen bond with the ligands (percentage). C) Hydrogen bond number in each complex as a function of time (10 ns). Abbreviation Hbond; hydrogen bond, ps; picosecond, SMO; smoothened protein, CLR; cholesterol, SNG; sonidegib.

Three trajectories each represents SMO-CLR, SMO-SNG and SMO-BST respectively, is independently simulated. This article shows how the structures and trajectories from molecular dynamics simulation can be used to analyse the interaction and energy contribution types throughout the simulation using available free software packages. Fig. 1 illustrates the details of hydrogen bonds between SMO and ligands using PyContact software [1]. Both SMO-CLR and SMO-BST show a similar pattern of hydrogen bond number. In contrast, all trajectories show no hydrophobic and salt-bridges formation between SMO and ligands (data not shown). Such data, when compared to *in vitro* findings, most of the residues that responsible for hydrogen bond have performed the expected role as previously reported [2], [3], [4].

**Fig. 2:**
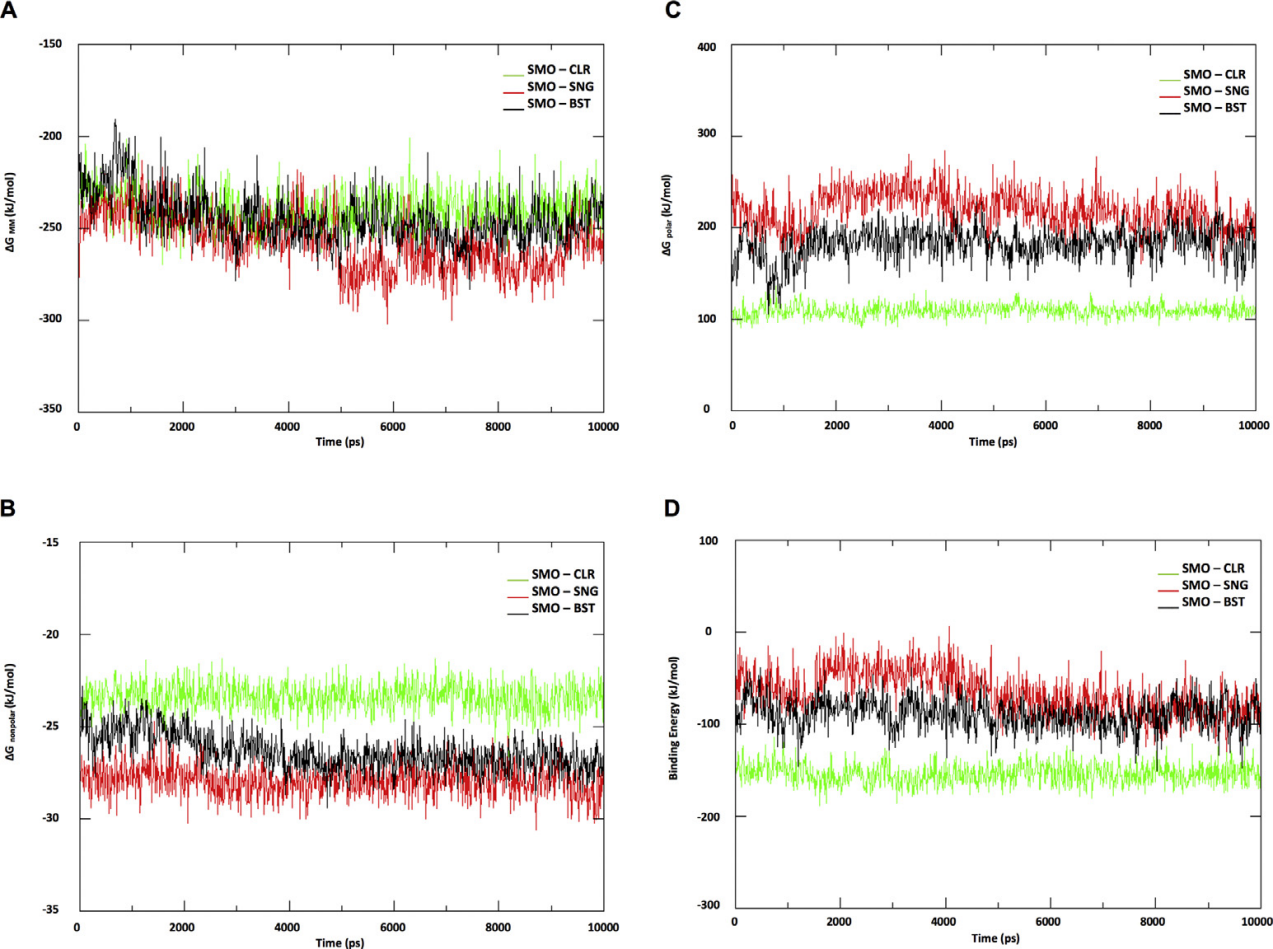
Energy components for protein-ligand complexes. A) The potential energy in the vacuum of complexes. B) Polar-solvation energy of complexes. C) Non-polar solvation energy of complexes. D) Binding energy combine of three energy components of complexes. Abbreviation ΔG; change in free energy, MM; molecular mechanics.

All-atom MD simulation in an explicit solvent of protein-ligand complexes produced various components of interaction energy that can be further manipulated by energy software tools to determine the binding energy or affinity of the protein-ligand. The structure and trajectory files of all complexes were subjected to analyses using g_mmpbsa tool [5]. The calculated energies were listed in Table 1. Each energy that contributes to binding energy or affinity is compared amongst each complex (Fig. 2). Surprisingly, the screening molecule β-sitosterol, having the lower binding energy compared with sonidegib by comprising lower polar solvation energy than sonidegib. However, when comparing all together, cholesterol contains the highest affinity against smoothened, which as expected since the cholesterol is an endogenous agonist for smoothened [6,7]. The .dat files which used for generation of the average energies values as listed in Table 1 are provided in Mendeley Data repository (http://dx.doi.org/10.17632/v94vzbwzf3.1).

**Table 1:** Summary of the energy of complexes.

**Energy**	**Complexes**	**Values (kJ/mol)**	**Standard deviation (kJ/mol)**
van der Waal	SMO-CLR	−209.794	+/- 10.771
	SMO-SNG	−226.937	+/- 11.996
	SMO-BST	−225.434	+/- 11.176
Electrostatic	SMO-CLR	−30.180	+/- 4.131
	SMO-SNG	−30.520	+/- 10.518
	SMO-BST	−19.259	+/- 9.100
Polar solvation	SMO-CLR	109.107	+/- 6.814
	SMO-SNG	219.131	+/- 21.175
	SMO-BST	182.092	+/- 18.136
SASA	SMO-CLR	−23.384	+/- 0.762
	SMO-SNG	−27.931	+/- 0.854
	SMO-BST	−26.375	+/- 0.997
SAV	SMO-CLR	0.000	+/- 0.000
	SMO-SNG	0.000	+/- 0.000
	SMO-CLR	0.000	+/- 0.000
WCA	SMO-CLR	0.000	+/- 0.000
	SMO-SNG	0.000	+/- 0.000
	SMO-CLR	0.000	+/- 0.000
Binding	SMO-CLR	−154.250	+/- 11.404
	SMO-SNG	−66.257	+/- 23.515
	SMO-BST	−88.976	+/- 17.117

kJ/mol; kilo Joule/mole, SASA; solvent-accessible surface area, SAV; Surface-area-to-volume ratio, WCA; Weeks−Chandler−Andersen decomposition scheme.

**Fig. 3:**
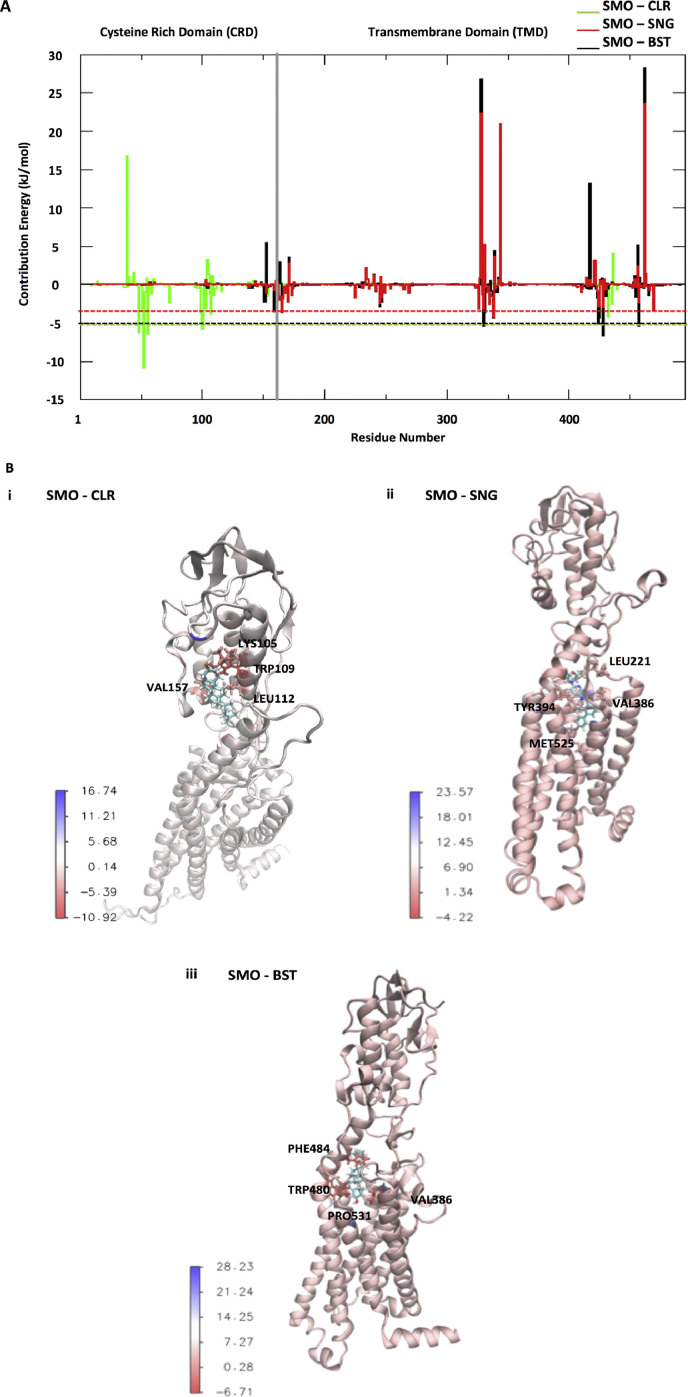
The energy contribution of smoothened amino acid residues. A) Energies of smoothened amino acid residues when binding with ligands. Residues number 1–165 represent residue 57–221 of CRD and 165–495 represent residue 222–551 of TMD. B) Most favourable residues in complexes that are contributing to the binding energy.

In order to identify the hotspot residues of smoothened protein-ligand binding, the contribution energy of each residue was explored. Energy decomposition of MM-PBSA was used to retrieve the individual amino acid contribution to the binding energy that assume to be crucial in the active site interacting residues (Fig. 3). The most favourable residues in SMO-CLR complex is TRP109 in the cysteine-rich domain (CRD) of smoothened which contributes the energy with less than −10 kJ/mol. This finding is consistent with a maximal well depth −10 kJ/mol at transmembrane 2/3e, which is the site of cholesterol-binding [8]. Residue TYR394 in SMO-SNG complex contributes to the highest binding energy towards sonidegib with the energy about −3 kJ/mol. The increase of energy contribution of phenylalanine 484 residue (<−5) increase the binding ability of smoothened to β-sitosterol. The energy contribution per residues in SMO-BST complex is similar with previous molecular dynamic analysis of inhibitors against smoothened protein [9].

Monitoring the fate of few residues located in transmembrane six (TM6) and transmembrane seven (TM7) has been proposed as a critical indicator for activation and inactivation of smoothened. Arginine 451 (ARG451) residue and tryptophan 535 (TRP535) residues of human smoothened located in transmembrane six and transmembrane seven respectively are showing to form a lock state as smoothened is in the inactive form. However, the interaction will abolish or in unlock state when smoothened undergoing the active form [10,11]. The trajectories files were used to examine the distance between ARG451 and TRP535 as a function of time.

**Fig. 4:**
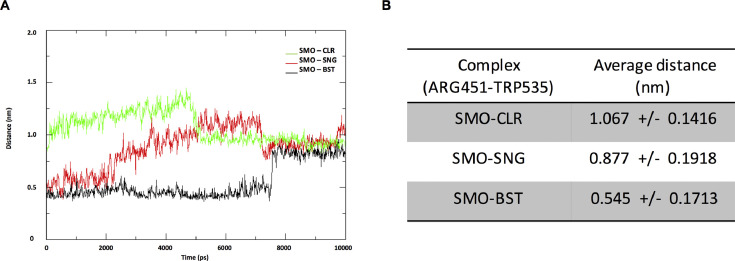
Distance measurement between side-chain of arginine 451 (ARG451) and tryptophan 535 (TRP535). A) All distance (nm) between side-chain of ARG451 and TRP535 for all complexes as a function of time (10 ns). B) Average distances of both residues in complexes as a function of time. Abbreviation: nm; nanometre.

Fig. 4 shows that side-chain of both residues in SMO-CLR complex maintains away about 1 nm throughout the simulation. Meanwhile, both residues in SMO-SNG complex show less distance compared with SMO-CLR. Interestingly, ARG451 and TRP535 residues in SMO-BST complex are kept stay close about 0.5 nm for eight ns of simulation. Supplementary video Figure S1, S2 and S3 for both residues state of SMO-CLR, SMO-SNG and SMO-BST, respectively as a function of time are supplemented in this article.

## 2 Experimental Design, Materials and Methods

### 2.1 MD simulation

MD simulation was carried out on complexes of Smoothened with ligands cholesterol, sonidegib and β-sitosterol using the GROMACS 2018.1 software. The ligand topology files were prepared by CGenFF, the official CHARMM general force field server while protein topology file was generated by GROMACS inbuilt command-line; gmx pdb2gmx with CHARMM36 all-atom force field. Both complexes were solvated in dodecahedron with the SPC/E water model, respectively. Four chloride ions were added to neutralise the charge of the system. The system energy was minimised using CHARMM36 force field without constraints using the steepest descent integrator for 50 000 steps, until a tolerance of 10 kJ/mol. After equilibrated at 300 K using V-rescale (modified Berendsen thermostat) for 100 ps as NVT ensemble, the system was then equilibrated at 1 atm pressure using Berendsen algorithm NPT ensemble for 100 ps. Finally, the MD simulation was carried out for 10 ns. The linear constraint solver (LINCS) algorithm, which is three to four times faster than the traditional SHAKE method, was used to constrain the length of covalent bonds. The particle-mesh Ewald summation technique was used to compute long-ranged electrostatic interactions. The Coulomb and van der Waal's cut-offs were set to 1.2 nm. During the MD simulation, the time step was defined as 2 fs. The coordinate trajectories were written at intervals of 10 ps.

### 2.2 Interaction types analysis

Using PyContact tool, interaction types between smoothened and cholesterol, sonidegib and β-sitosterol were analysed. The tool works on the GROMACS generated topology (.tpr) and trajectory (.xtc) files. PyContact tool was executed in the terminal by typing pycontact. The topology and trajectory files were loaded into the tool and selection 1 box was typed with segid seg_0_Protein_chain_A while selection 2 box with segid seg_1_ligand name. The OK button was clicked for the tool to start analyse the trajectory file. Once finished, accumulate score button was clicked, and resid and resname selection were checked for visualising the interaction between the selection (SMO and ligand). All interactions such as type of interactions, mean score, mean lifetime, Hbond percentage, Hbond number, timeline throughout the simulation time saved either as png or vector graphic format.

### 2.3 Calculation of binding energy and residue contribution energy

Using g_mmpbsa tool, the binding energy of cholesterol, sonidegib and β-sitosterol were calculated. g_mmpbsa tool works on the GROMACS generated structure (.tpr) and trajectory (.xtc) files and calculates mainly three components of the binding energy, *i.e.*, molecular mechanical energy, polar solvation energy and apolar solvation energy. For calculation of polar solvation energy, g_mmpbsa relies on Assisted Poison Boltzmann Solver (APBS) program. For apolar solvation energy calculation Solvent Accessible Surface Area (SASA) model was used. Molecular mechanical (MM) energy consisted of electrostatic contribution (Eelec) and van der Waals (EvdW) contributions. Binding free energy calculation relies on the following equation (I) and (II). Data files (.dat or .xvg) generated from the calculation were used to plot the graphs using Grace tools.

(I) ΔGbinding = Gcomplex − (Gprotein + Gligand)

where G term can be further decomposed into the following components

(II) ΔG = ΔEMM + ΔGsolvation − TΔS = ΔE(bonded+non-bonded) + ΔG(polar+non-polar) – TΔS

Residue wise free energy contribution was calculated with the help of MmPBSADecomp.py script provided along with the g_mmpbsa tool. The MmPBSADecomp.py script relies on the energetic terms obtained from the g_mmpbsa and calculates the average free energy contribution of each residue. The three energetic terms used for the calculation of residue wise free energy calculation were molecular mechanical (MM), polar solvation and apolar solvation. Binding free energy contributions are less than −0.1 kcal mol−1 and greater than 0.1 kcal mol−1 was shown in residues wise free energy decomposition diagrams. All frames covering the period of 10 ns of the stable MD trajectories were used for the binding free energy analysis of all complexes. Visualising of the most favourable (contribution energy) amino acid residues was done using VMD and were save as PNG format.

### 2.4 Determine the distance between ARG451 and TRP535

The distance between ARG451 and TRP535 of smoothened protein in all complexes as a function of time were measured using GROMACS 2018 command-line; gmx distance. Group of side-chain of ARG451 and TRP535 were created in the GROMACS index file, and these groups were chosen for calculation of the distance. The gromacs file (.gro) for all complexes were generated from both structure and trajectory files using command-line; gmx trjconv, and visualization of the distance between pair was carried out using VMD. Movies in mpg format were generated from VMD and further converted into mp4 format online.

## Ethics Statement

No humans and animals were involved in the data analysis.

## Declaration of Competing Interest

The author declares that no known competing financial interests or personal relationships which have, or could be perceived to have, influenced the work reported in this article.

## Appendix Supplementary materials

Fig. S1. Supplementary video 1 Movie of MD trajectory showing the state of side-chain of ARG451 and TRP535 for SMO-CLR complex within 10 ns of simulation Image, video 1

Fig. S2. Supplementary video 2 Movie of MD trajectory showing the state of side-chain of ARG451 and TRP535 for SMO-SNG complex within 10 ns of simulation Image, video 2

Fig. S3. Supplementary video 3 Movie of MD trajectory showing the state of side-chain of ARG451 and TRP535 for SMO-BST complex within 10 ns of simulation Image, video 3

Image

Image

Image
