# Supplementary figures and images for: Data analysis of molecular dynamics simulation trajectories of β-sitosterol, sonidegib and cholesterol in smoothened protein with the CHARMM36 force field
Source: Data Brief. 2020 Sep 29;33:106350. doi: 10.1016/j.dib.2020.106350 (PMC7554031; doi:10.1016/j.dib.2020.106350)

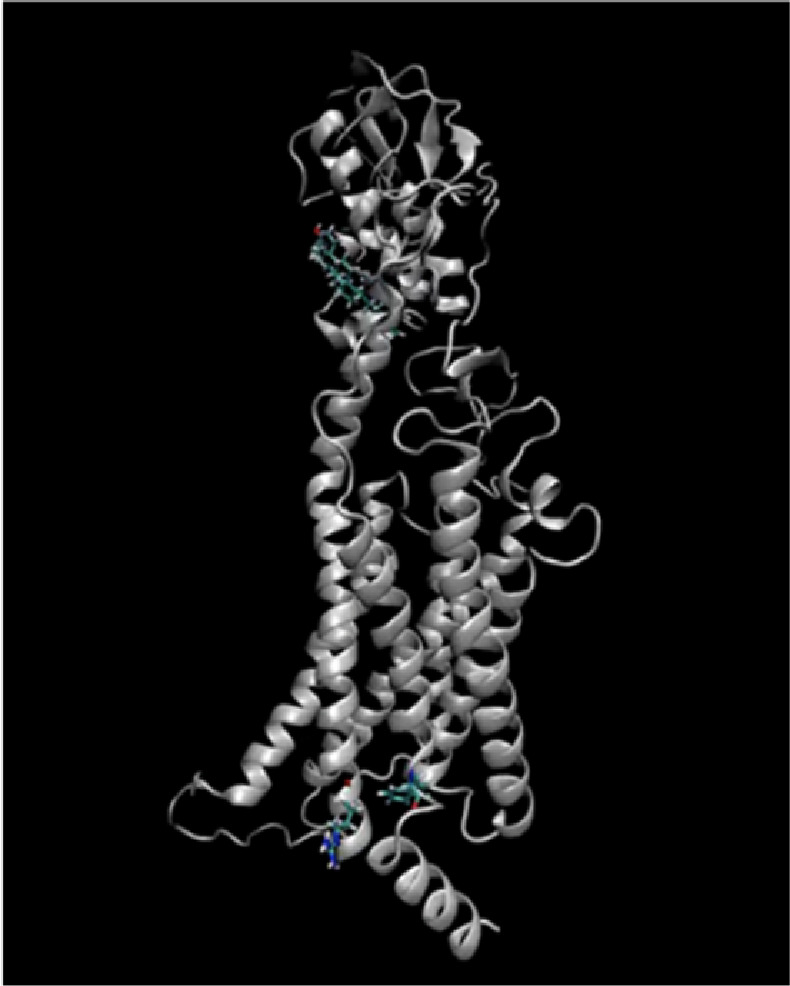

Supplement: Supplementary file 4 [file mmc4.jpg]

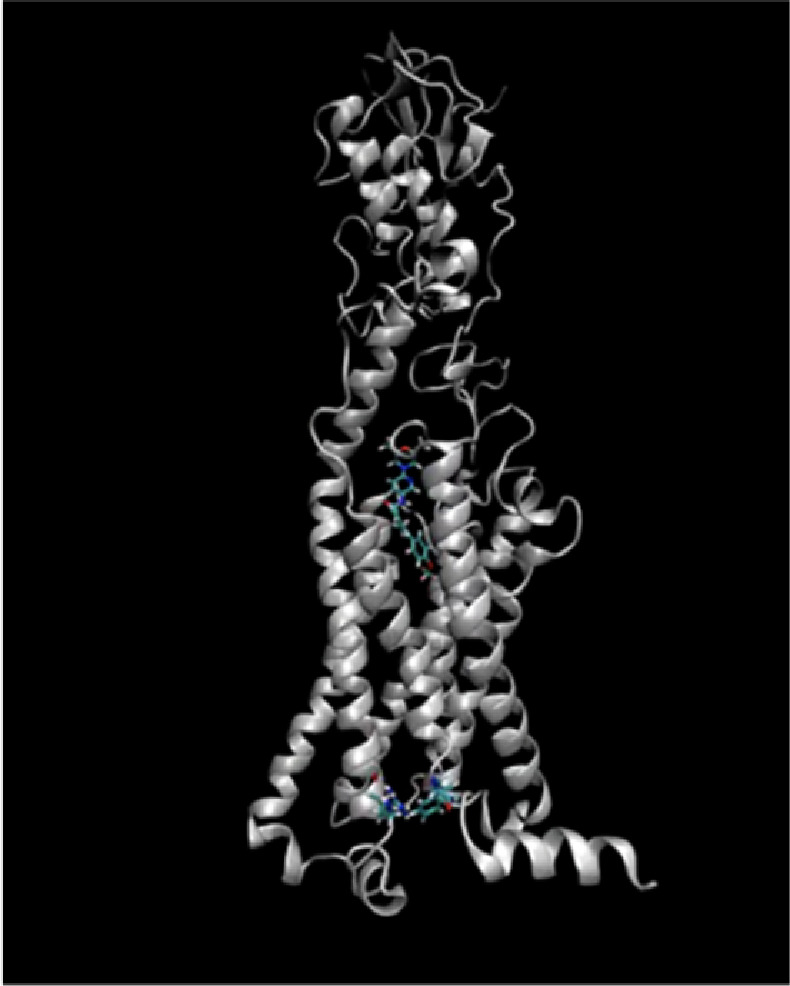

Supplement: Supplementary file 5 [file mmc5.jpg]

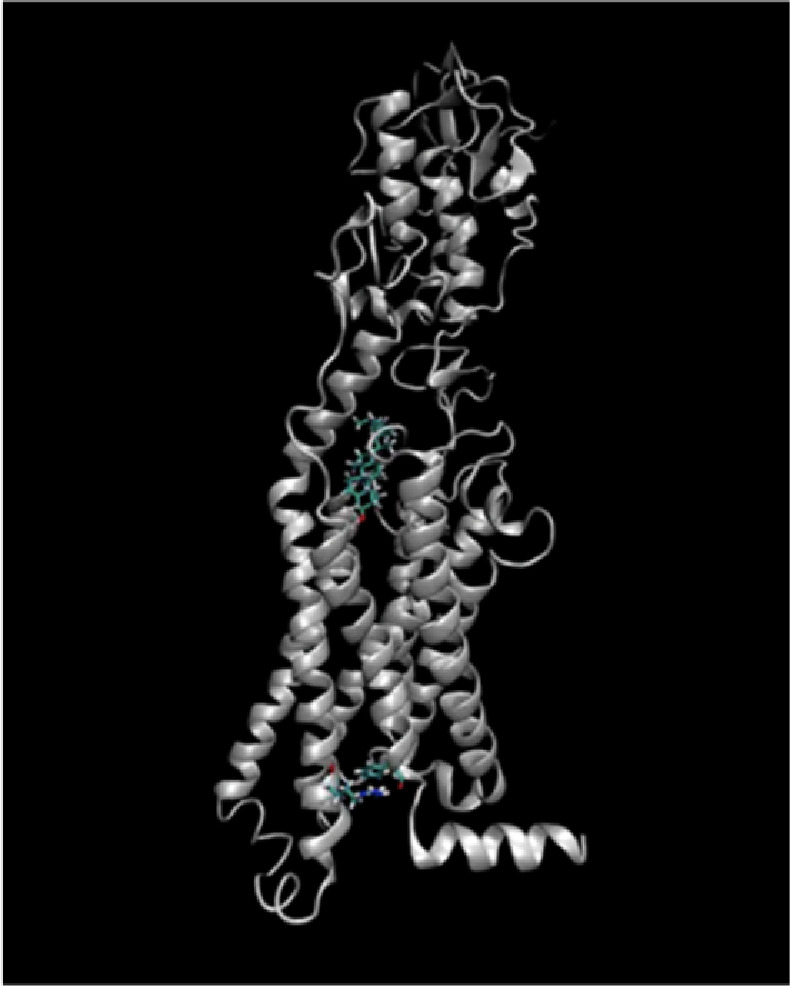

Supplement: Supplementary file 6 [file mmc6.jpg]
